# Supplementary figures and images for: Drug-target binding quantitatively predicts optimal antibiotic dose levels in quinolones
Source: PLoS Comput Biol. 2020 Aug 14;16(8):e1008106. doi: 10.1371/journal.pcbi.1008106 (PMC7449454; doi:10.1371/journal.pcbi.1008106)

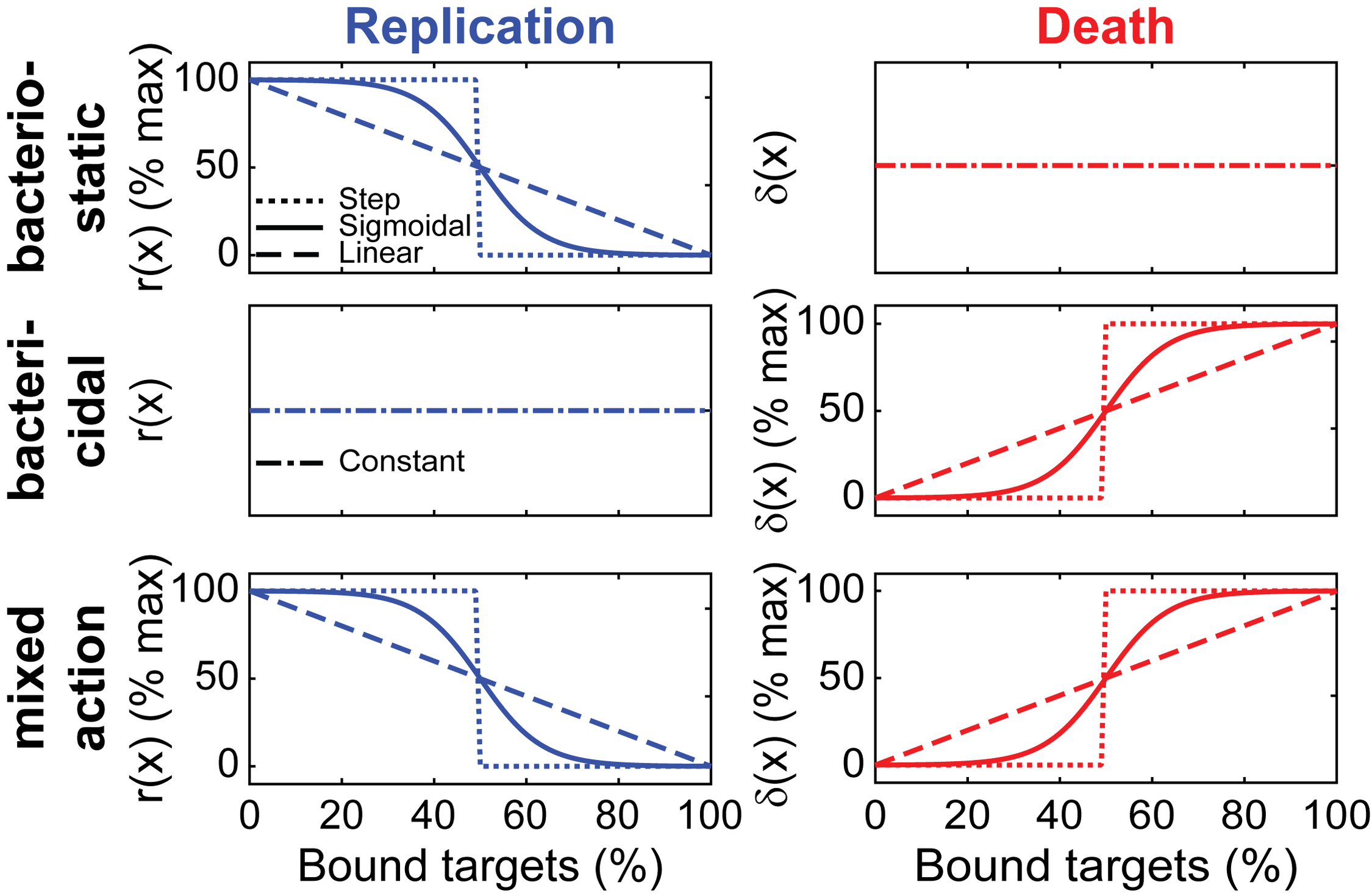

Supplement: S1 Fig — These graphs show which functions were used to fit the dependence of bacterial replication, r(x), and death, δ(x), on target occupancy for antibiotics with bacteriostatic, bactericidal or mixed action. Solid lines indicate a sigmoidal relationship, dotted lines indicate a step function, dashed lines indicate a linear relationship, and dash-dotted lines indicate independence, i.e. a constant replication or death rate. The left panels, show the replication rates (blue), the right panels show the death rates (red). The top panels, show rates for a bacteriostatic drug, the middle panels, show rates for a bactericidal drug, and the bottom panels, show rates for a drug with mixed effects. The sum of the replication and death rates at certain target occupancies gives the net growth or decline rate of the bacterial population. (TIF) [file pcbi.1008106.s001.tif]

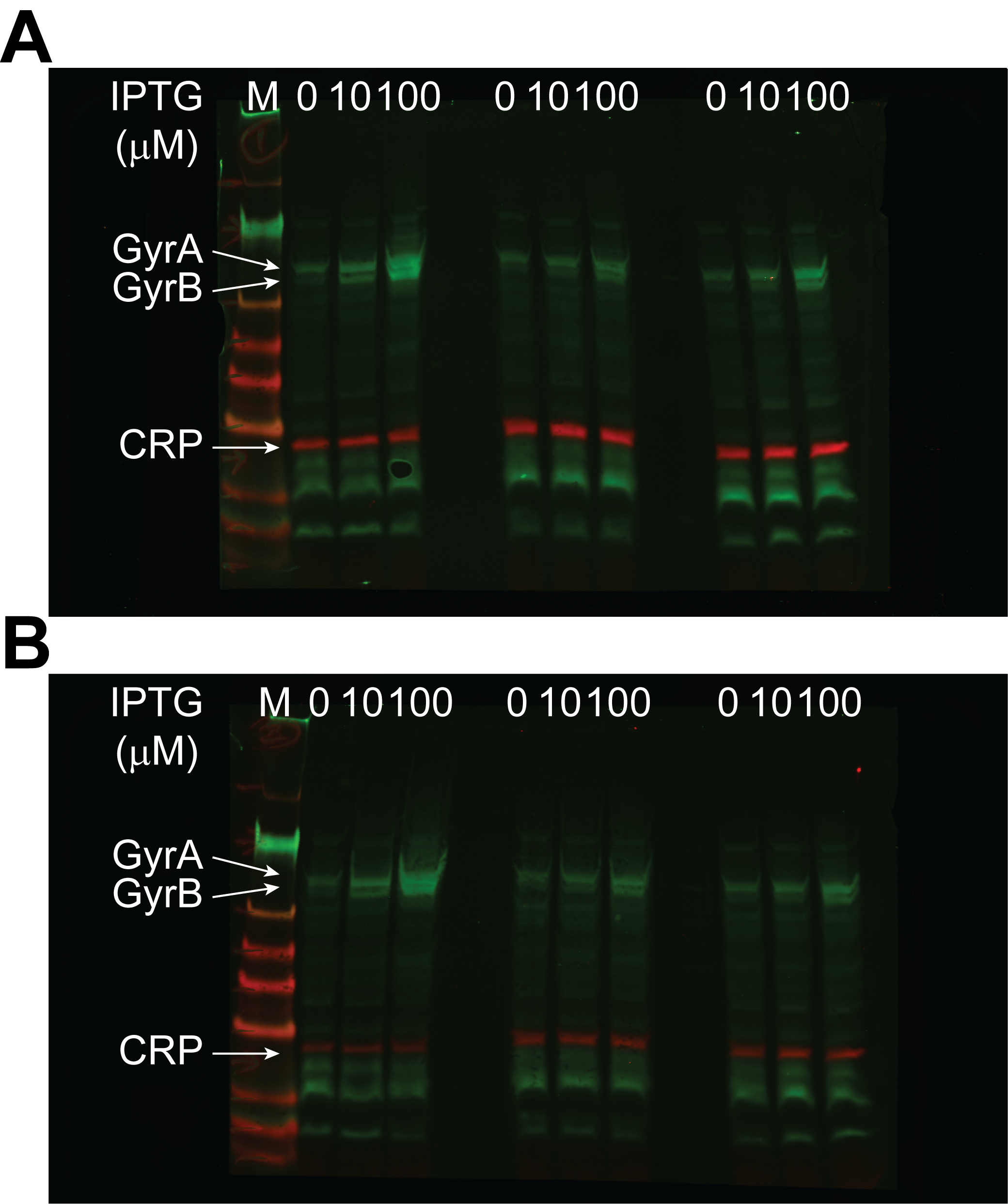

Supplement: S2 Fig — a, b, E. coli expressing gyrA and gyrB under control of the same IPTG-inducible promoter (SoA3329) grown in the presence of 10 μM IPTG (mild overexpression) and 100 μM IPTG (strong overexpression). A control strain containing a mock plasmid (SoA3330), representing wild-type GyrAB levels, was grown in the absence of inducer. Whole cell lysates were separated on a SDS-PAGE gel, blotted, and detected with specific fluorescent antibodies against GyrA (green), GyrB (green) and CRP (red). CRP (cAMP receptor protein) was used as loading control. (TIF) [file pcbi.1008106.s002.tif]

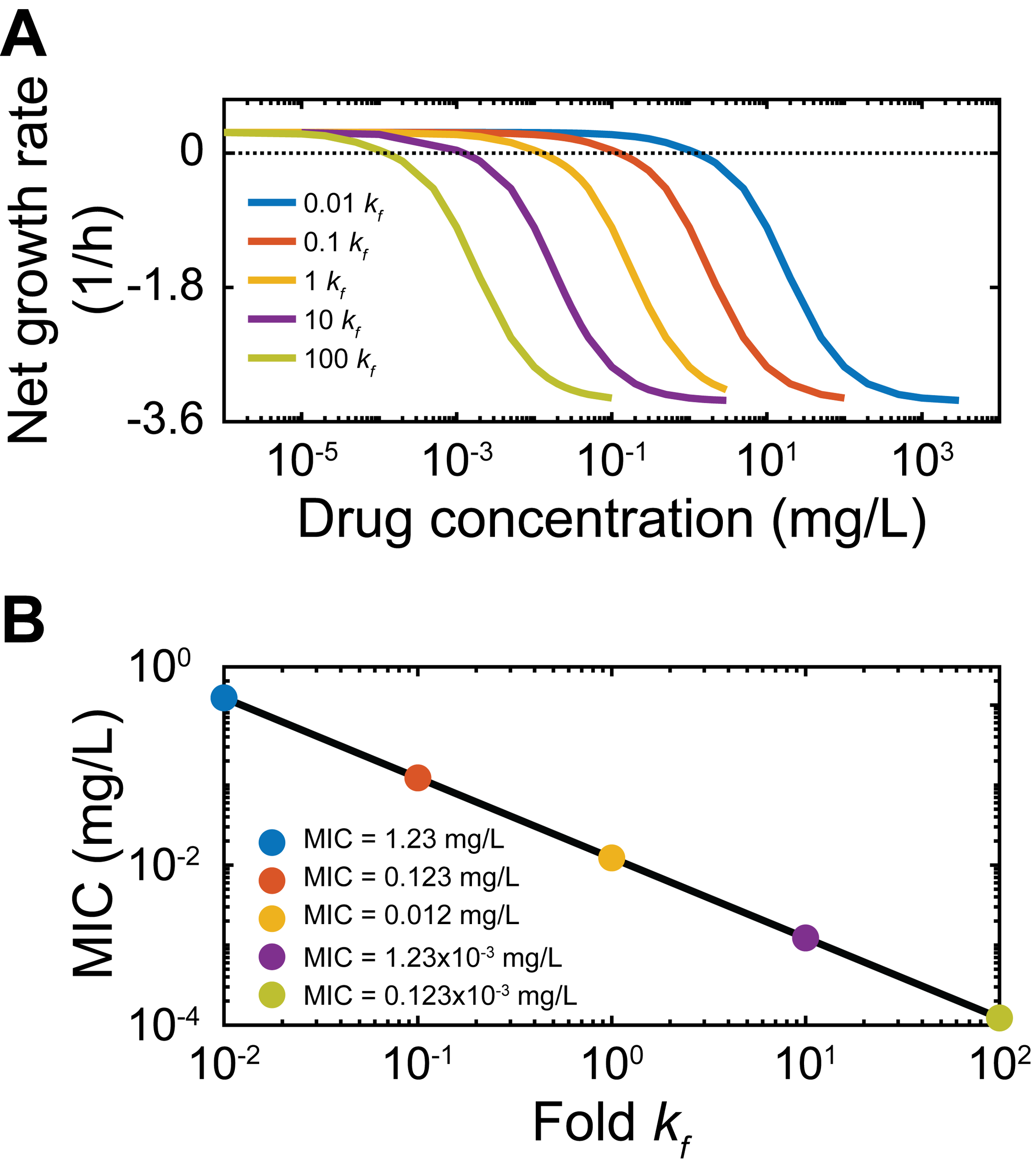

Supplement: S3 Fig — We use the model fitted to experimental data to explore the sensitivity of our results to changes in kf (0.01x, 0.1x, 1x, 10x, and 100x original value). a, Net growth rate (log10(bacterial number at 18 h)—log10(bacterial number at 0 h))/18 h) as function of drug concentration for different values of the binding rate kf (see legend). The dotted horizontal line indicates zero net growth. The intersections of the simulated dose-response curves with this line indicate the corresponding MICs. b, Sensitivity of the MIC to kf obtained from simulations in (a). The color code indicates the MIC corresponding to the simulation with the same color in (a). (TIF) [file pcbi.1008106.s003.tif]

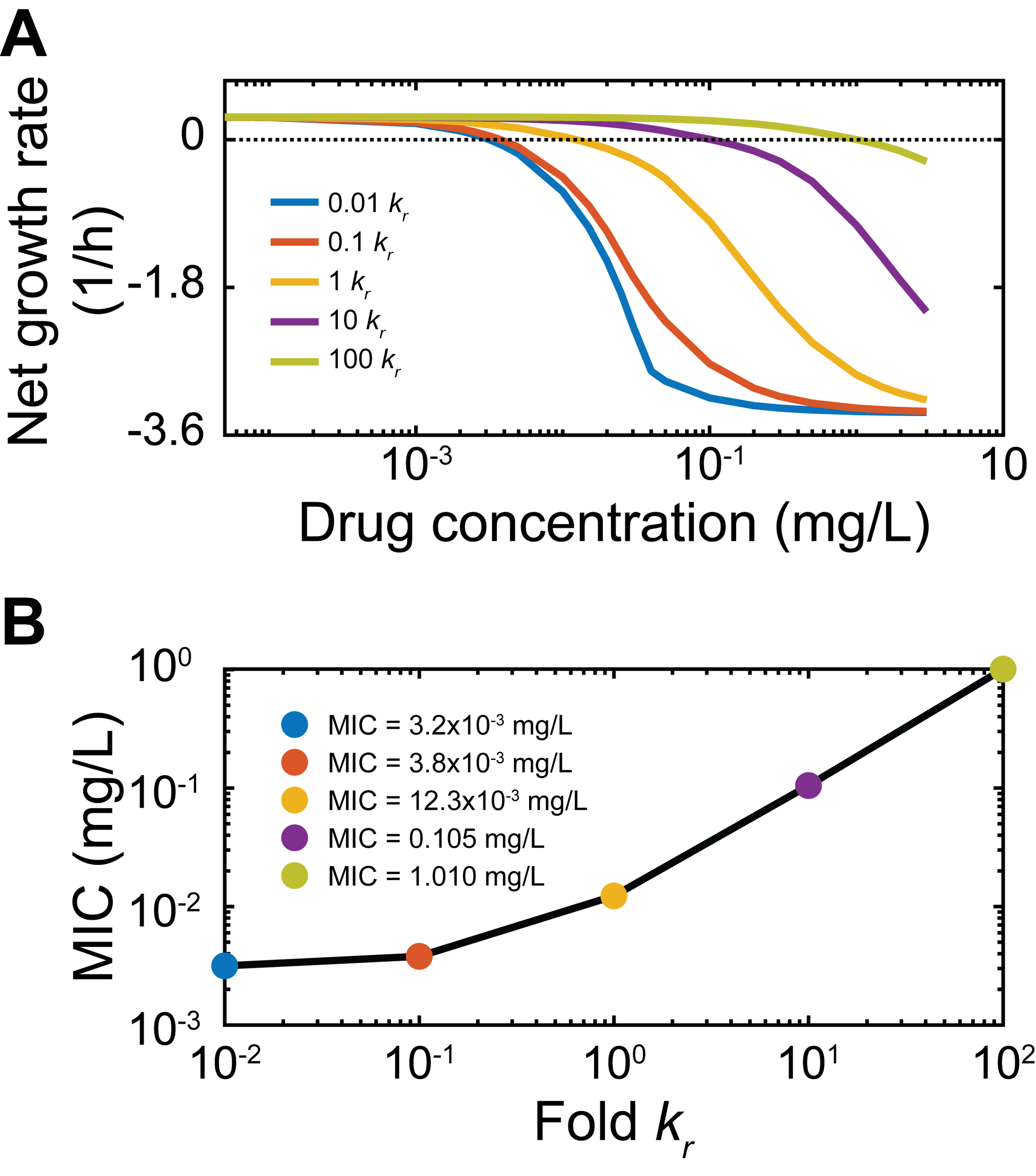

Supplement: S4 Fig — We use the model fitted to experimental data to explore the sensitivity of our results to changes in kr (0.01x, 0.1x, 1x, 10x, and 100x original value). a, Net growth rate (log10(bacterial number at 18 h)—log10(bacterial number at 0 h))/18 h) as function of drug concentration for different values of the binding rate kr (see legend). The dotted horizontal line indicates zero net growth. The intersections of the simulated dose-response curves with this line indicate the respective MICs. b, Sensitivity of the MIC to kr obtained from simulations in (a). The color code indicates the MIC corresponding to the simulation with the same color in (a). (TIF) [file pcbi.1008106.s004.tif]

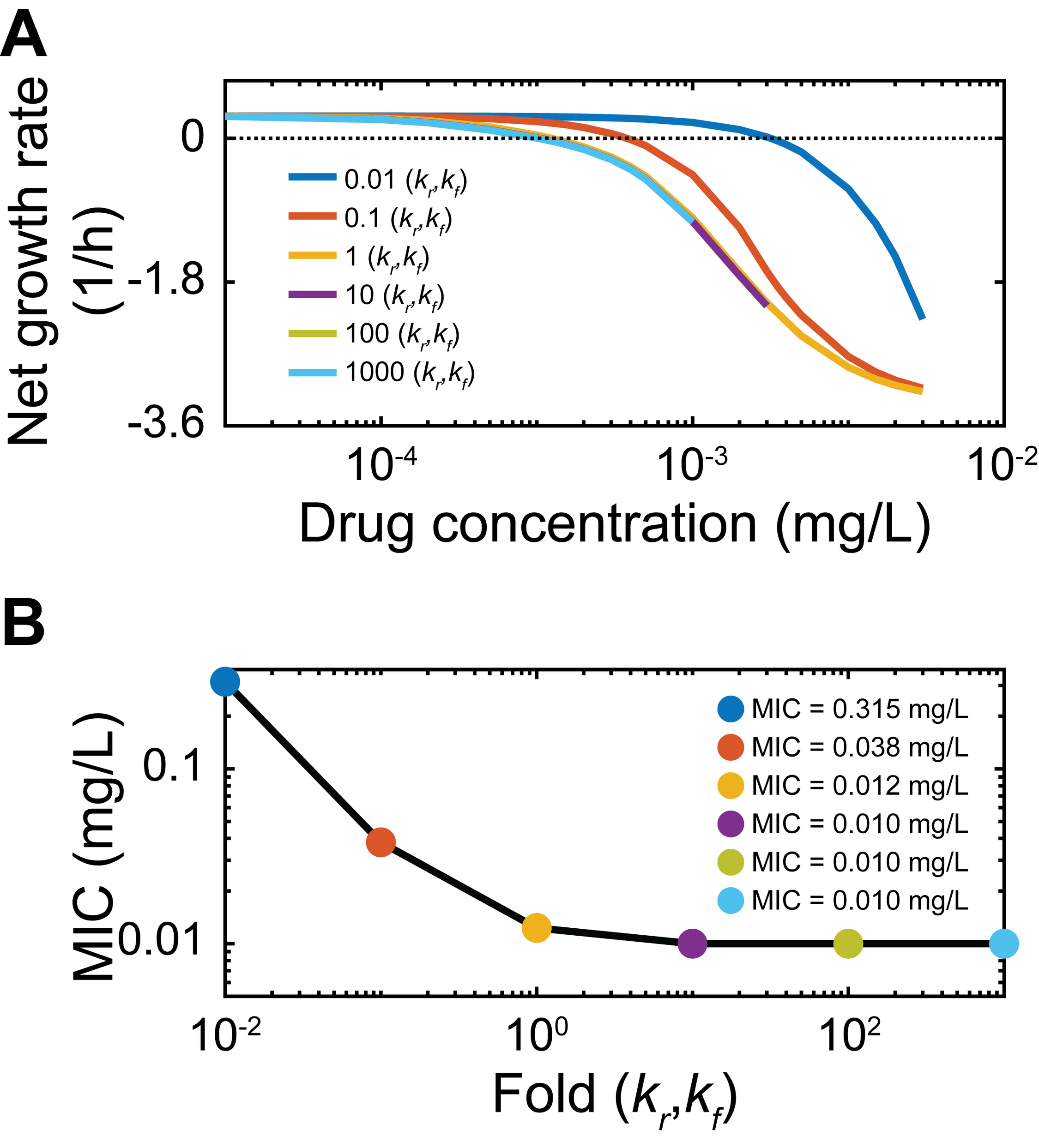

Supplement: S5 Fig — We use the model fitted to experimental data to explore the sensitivity of our results to changes in the turnover rate of the drug-target complex. We changed kr and kf (0.01x, 0.1x, 1x, 10x, 100x, and 1000x original value) while keeping the ratio between kf and kr, the affinity KD, constant. a, shows the net growth rate (log10(bacterial number at 18 h)—log10(bacterial number at 0 h))/18 h) as function of drug concentration for different values of the turnover rate. The dotted horizontal line indicates zero net growth. The intersections of the simulated dose-response curves with this line indicate the respective MICs. b, Sensitivity of the MIC to turnover rate obtained from simulations in (a). The color code indicates the MIC corresponding to the simulation with the same color in (a). (TIF) [file pcbi.1008106.s005.tif]

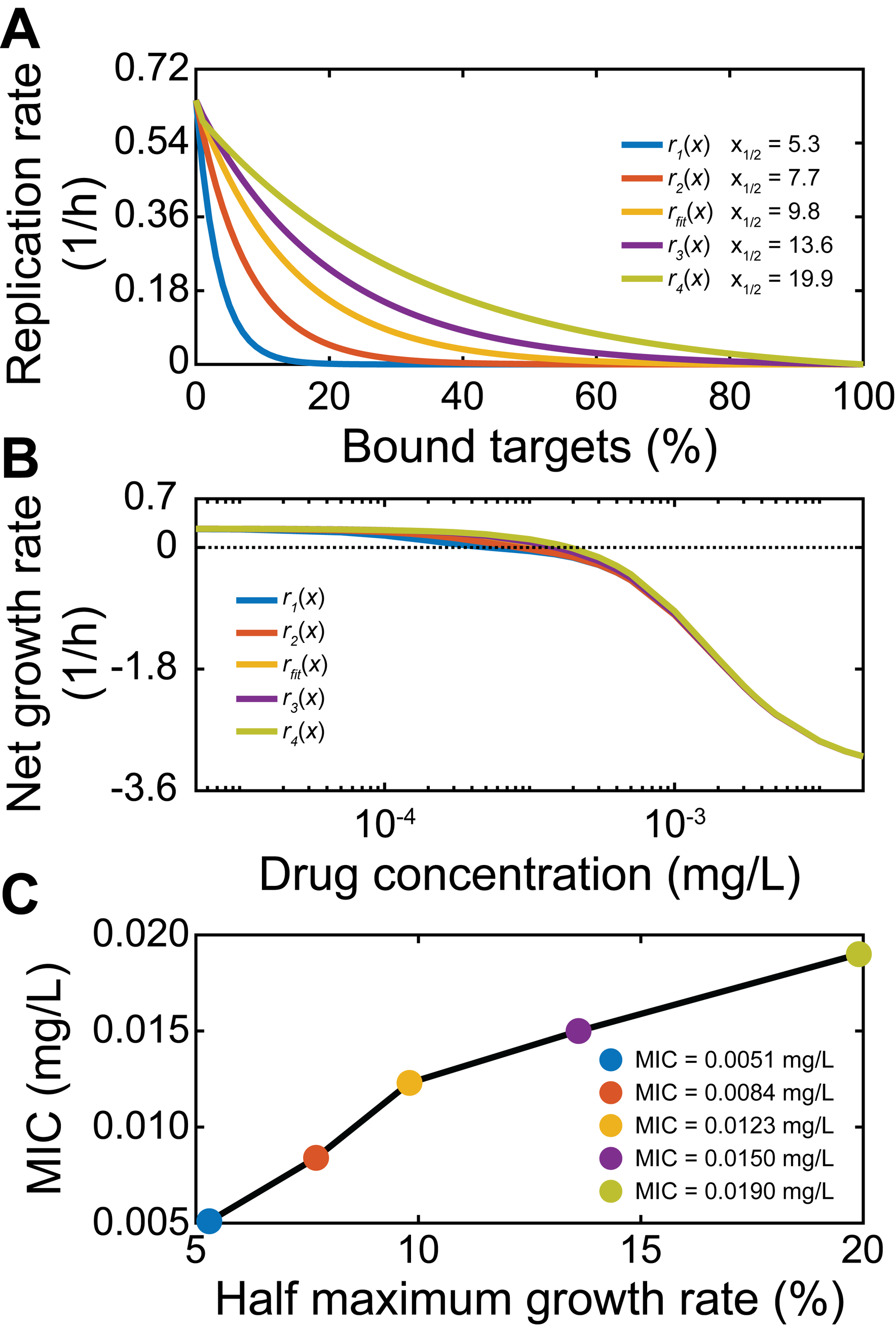

Supplement: S6 Fig — We use the model fitted to experimental data to explore the sensitivity of our results to changes in the replication rate with increasingly bound target r(x). We change the value of bound target at which we obtain a half-maximal replication rate, x1/2. a, Functions connecting bacterial replication rates r(x) to percentage of bound target molecules with different half-maximal replication rates. b, Net growth rate (log10(bacterial number at 18 h)—log10(bacterial number at 0 h))/18 h) as function of drug concentration for different values of x1/2 (see legend). The dotted horizontal line indicates zero net growth. The intersections of the simulated dose-response curves with this line indicate the respective MICs. c, Sensitivity of the MIC to r(x) obtained from simulations in (b). The color code indicates the MIC corresponding to the simulation with the same color in (a&b). (TIF) [file pcbi.1008106.s006.tif]

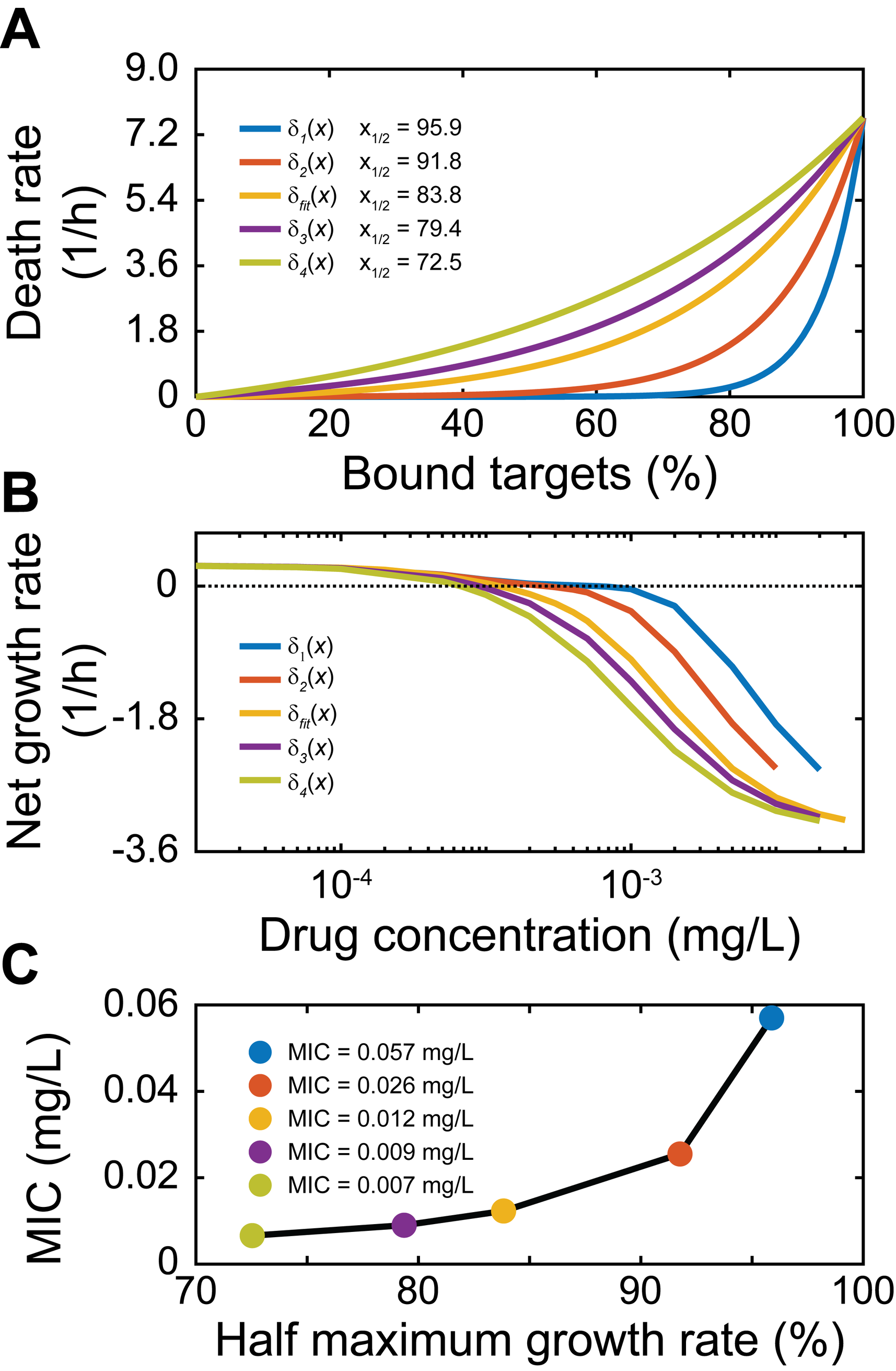

Supplement: S7 Fig — We use the model fitted to experimental data to explore the sensitivity of our results to changes in the death rate with increasingly bound target δ(x). We change the value of bound target at which we obtain a half-maximal death rate, x1/2. a, Functions connecting bacterial death rates δ(x) to percentage of bound target molecules with different half-maximal death rates. b, Net growth rate (log10(bacterial number at 18 h)—log10(bacterial number at 0 h))/18 h) as function of drug concentration for different values of x1/2 (see legend). The dotted horizontal line indicates zero net growth. The intersections of the simulated dose-response curves with this line indicate the respective MICs. c, Sensitivity of the MIC to δ(x) obtained from simulations in (b). The color code indicates the MIC corresponding to the simulation with the same color in (a&b). (TIF) [file pcbi.1008106.s007.tif]

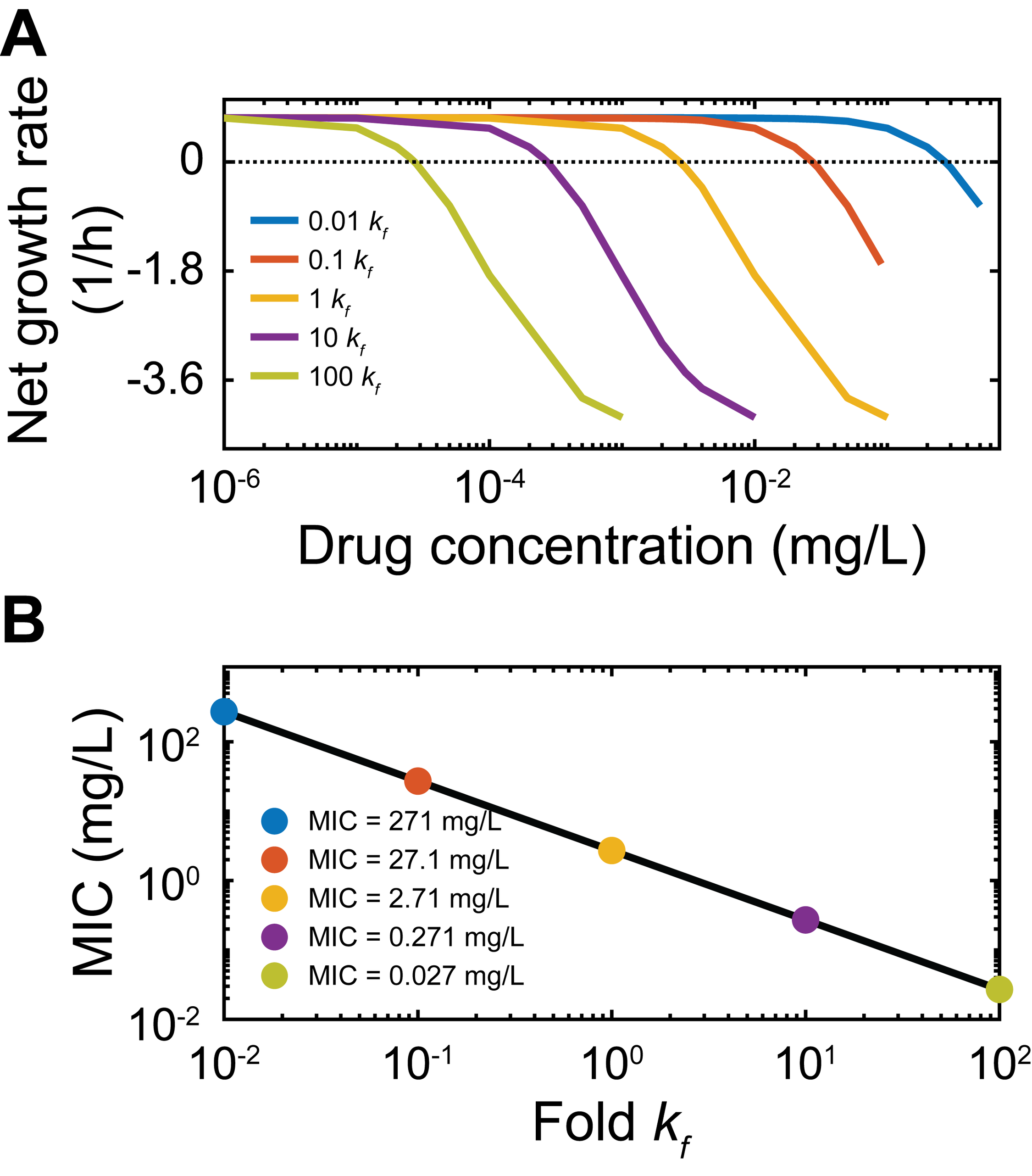

Supplement: S8 Fig — We use the model fitted to experimental data (Fi 5) to explore the sensitivity of our results to changes in kf (0.01x, 0.1x, 1x, 10x, and 100x original value). a, Net growth rate (log10(bacterial number at 18 h)—log10(bacterial number at 0 h))/18 h) as function of drug concentration for different values of the binding rate kf (see legend). The dotted horizontal line indicates zero net growth. The intersections of the simulated dose-response curves with this line indicate the respective MICs. b, Sensitivity of the MIC to kf obtained from simulations in (a). The color code indicates the MIC corresponding to the simulation with the same color in (a). (TIF) [file pcbi.1008106.s008.tif]

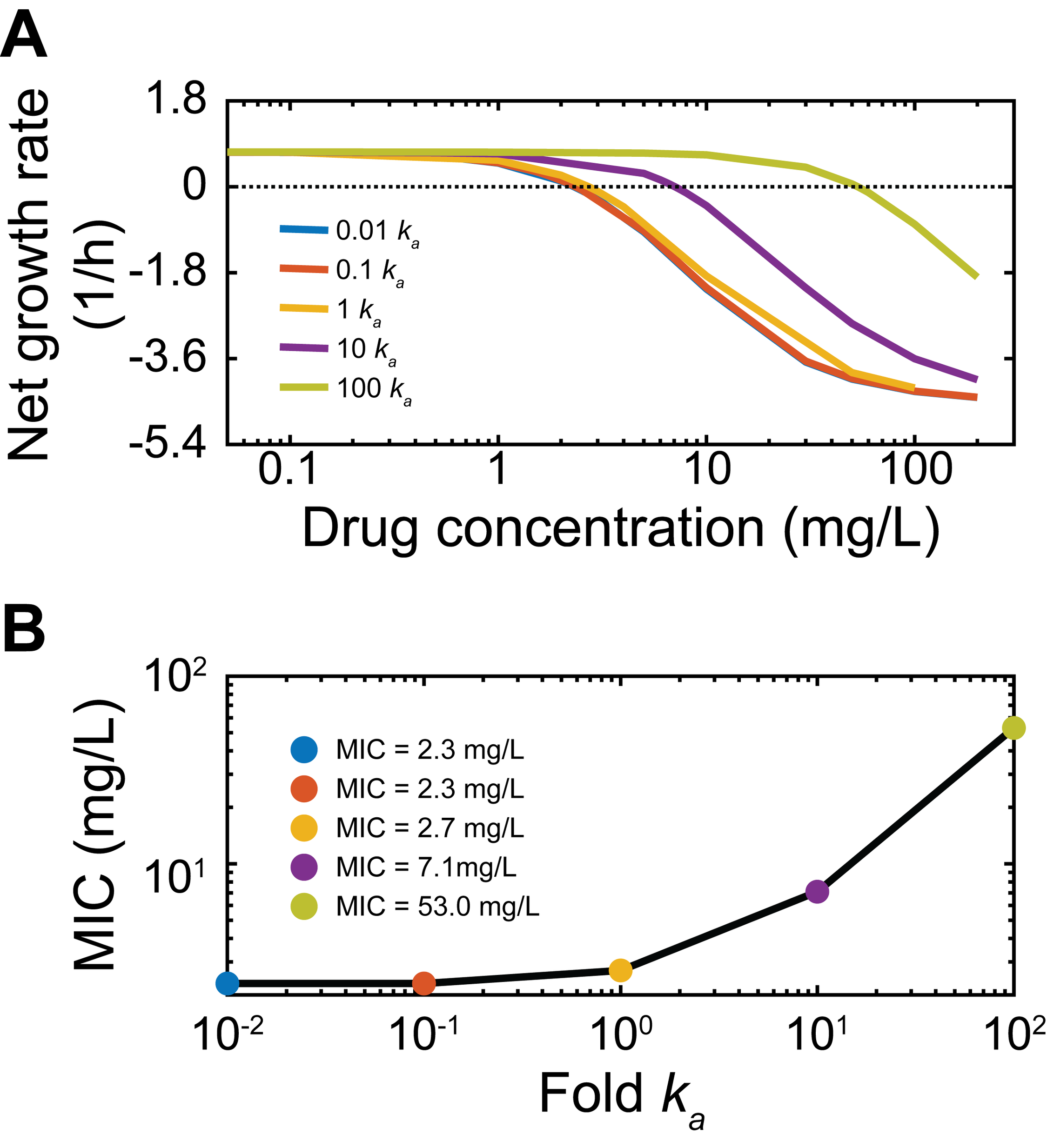

Supplement: S9 Fig — We use the model fitted to experimental data (Fig 5) to explore the sensitivity of our results to changes in ka (0.01x, 0.1x, 1x, 10x, and 100x original value). a, Net growth rate (log10(bacterial number at 18 h)—log10(bacterial number at 0 h))/18 h) as function of drug concentration for different values of the binding rate ka (see legend). The dotted horizontal line indicates zero net growth. The intersections of the simulated dose-response curves with this line indicate the respective MICs. b, Sensitivity of the MIC to ka obtained from simulations in (a). The color code indicates the MIC corresponding to the simulation with the same color in (a). (TIF) [file pcbi.1008106.s009.tif]

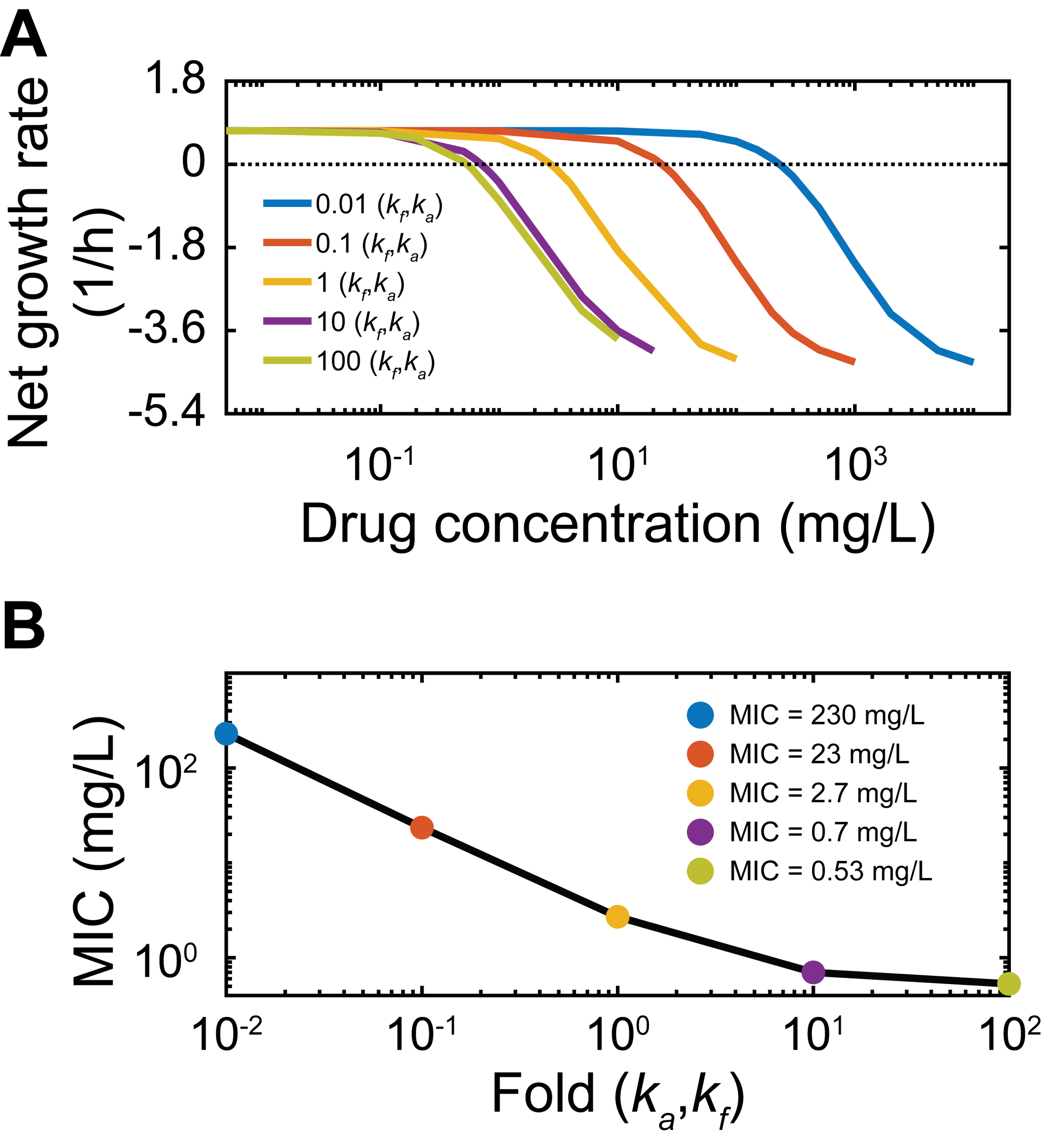

Supplement: S10 Fig — We use the model fitted to experimental data (Fig 5) to explore the sensitivity of our results to changes in the turnover rate of the drug-target complex. We changed values for ka and kf (0.01x, 0.1x, 1x, 10x, and 100x original value) while keeping the ration of ka/kf constant. a, Net growth rate (log10(bacterial number at 18 h)—log10(bacterial number at 0 h))/18 h) as function of drug concentration for different values of the turnover rate (see legend). The dotted horizontal line indicates zero net growth. The intersections of the simulated dose-response curves with this line indicate the respective MICs. b, Sensitivity of the MIC to turnover rate obtained from simulations in (a). The color code indicates the MIC corresponding to the simulation with the same color in (a). (TIF) [file pcbi.1008106.s010.tif]

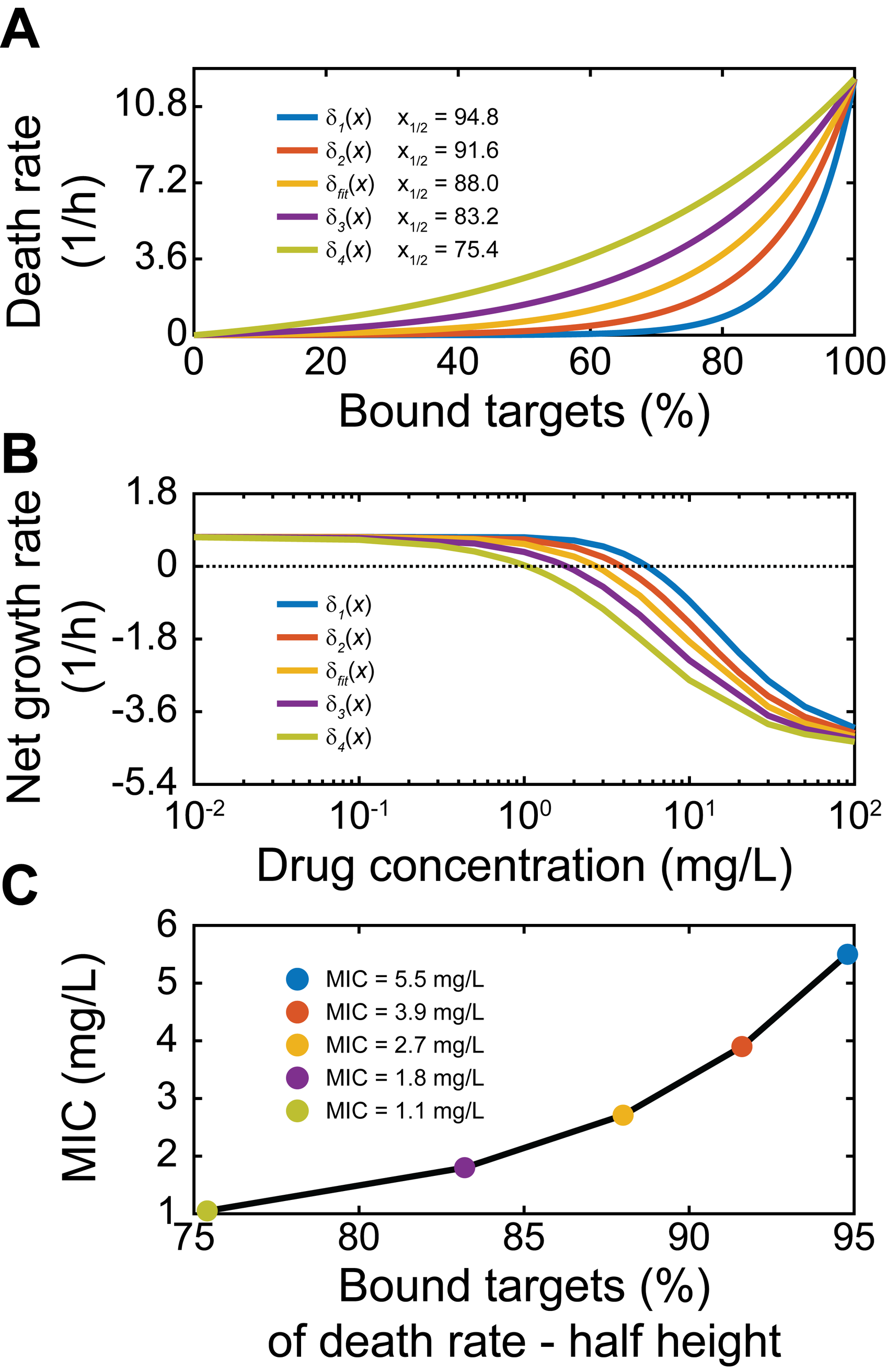

Supplement: S11 Fig — We use the model fitted to experimental data (Fig 5) to explore the sensitivity of our results to changes in the death rate with increasingly bound target δ(x). We change the value of bound target at which we obtain a half-maximal death rate, x1/2 (see legend). a, Functions connecting bacterial death rates δ(x) to percentage of bound target molecules with different half-maximal replication rates x1/2 (see legend). b, Net growth rate (log10(bacterial number at 18 h)—log10(bacterial number at 0 h))/18 h) as function of drug concentration for different values of δ(x) (see legend). The dotted horizontal line indicates zero net growth. The intersections of the simulated dose-response curves with this line indicate the respective MICs. c, Sensitivity of the MIC to δ(x) obtained from simulations in (b). The color code indicates the MIC corresponding to the simulation with the same color in (a&b). (TIF) [file pcbi.1008106.s011.tif]

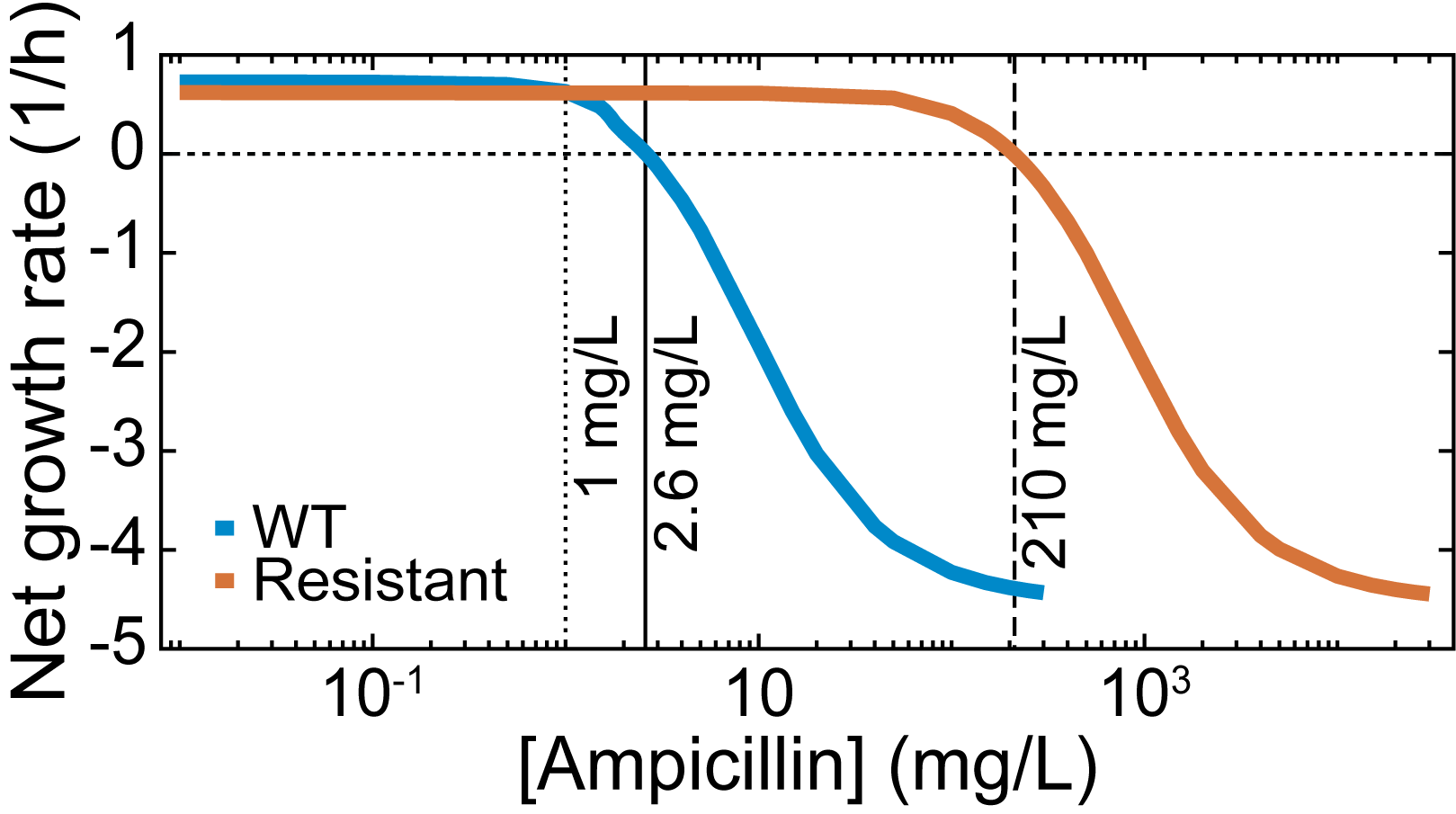

Supplement: S12 Fig — The drug concentration of ampicillin is shown on the x-axes, and the average bacterial net growth rate over 18 h is given on the y-axes. The blue line represents the wild-type strain based on the fits shown in Fig 5, and the red line represents a strain with a theoretical resistance mutation that decreases the binding rate (kf) 100-fold and imparts a 15% fitness cost. The dotted horizontal line represents no net growth. The first vertical dotted line indicates where the resistant strain becomes fitter than the wild-type (the start of the competitive resistance selection window), the solid vertical line indicates the MIC of the wild-type (the start of the classical resistance selection window), and the dashed vertical line indicates the MIC of the resistant strain, above which selection for resistance should be minimal because both growth of the wild-type and the resistant strain is inhibited. (TIF) [file pcbi.1008106.s012.tif]

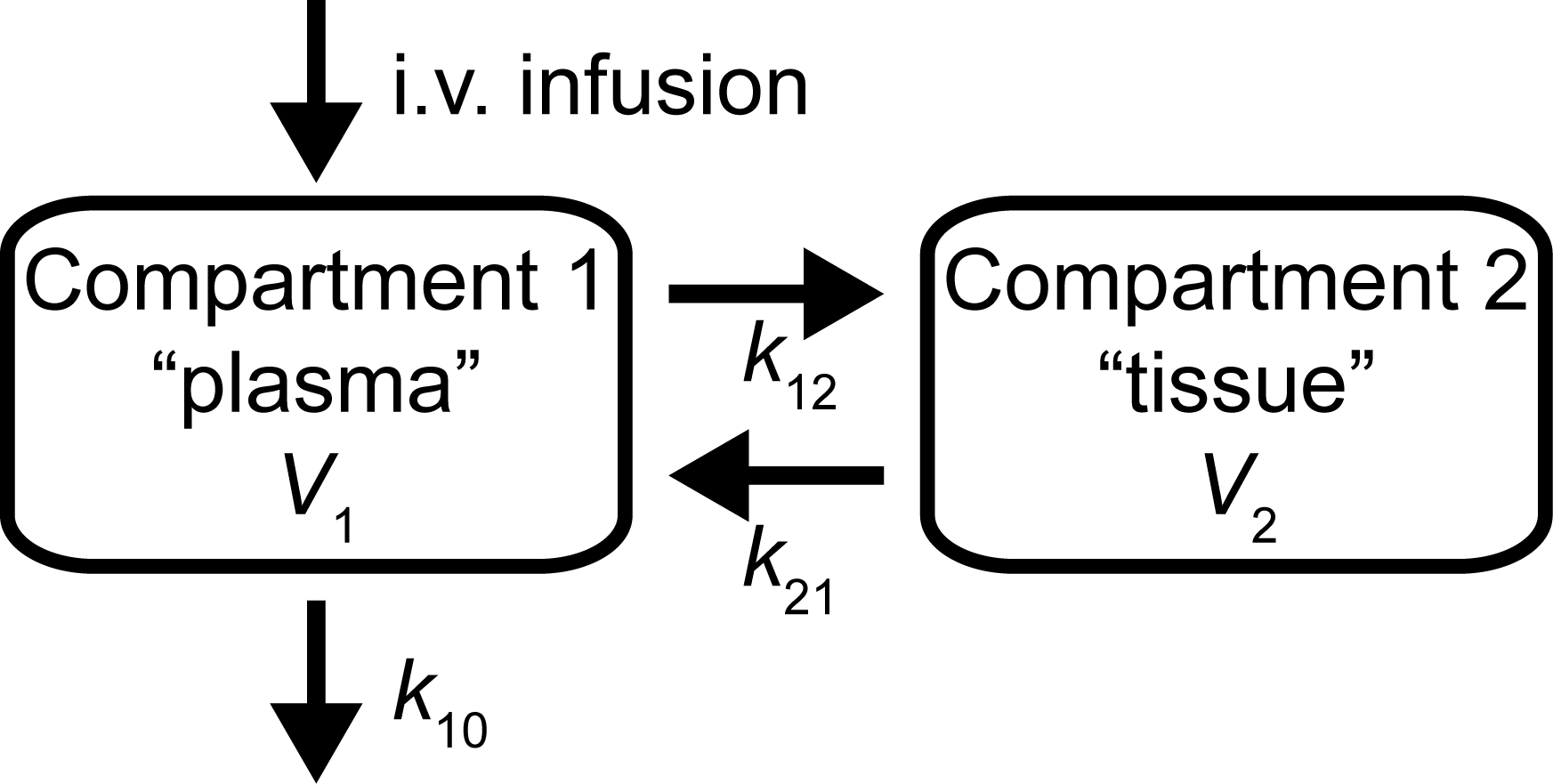

Supplement: S13 Fig — We simulate plasma and tissue concentrations of ampicillin with a two-compartment pharmacokinetic model. This model described intravenous drug input into the “plasma” compartment, which has an apparent volume of V1. From there, it can enter the peripheral “tissue” compartment, characterized by the apparent volume V2, with a rate k12. Conversely, the drug can also re-enter the plasma compartment with a rate k21. From the plasma compartment, the drug is eliminated with a rate k10. (TIF) [file pcbi.1008106.s013.tif]

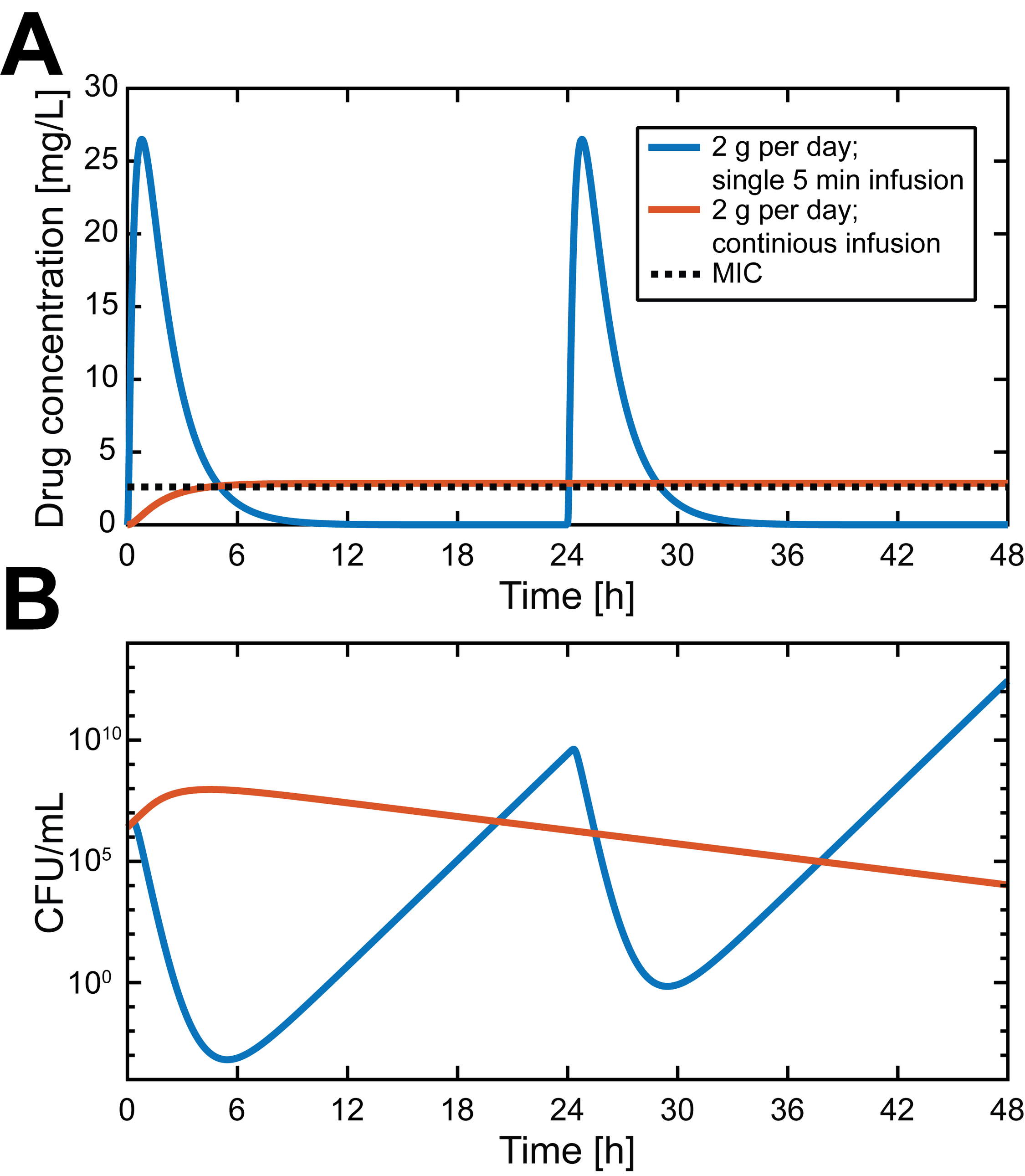

Supplement: S14 Fig — Two modes of drug administrations, 2 g of drug per day given as single, 5 min i.v. infusions (blue line) and 2 g of drug per day given as continuous i.v. infusion (red line), are simulated. a, Simulated drug concentrations in the tissue (i.e., infected) compartment of a two compartment pharmacokinetic model over two days. The dotted black line indicates the MIC of the pathogen (2.6 mg/L). b, Pathogen load in the tissue compartment in response to fluctuating drug concentrations predicted by COMBAT over the same timeframe. (TIF) [file pcbi.1008106.s014.tif]
