# Supplementary material for: Drug-target binding quantitatively predicts optimal antibiotic dose levels in quinolones
Source: PLoS Comput Biol. 2020 Aug 14;16(8):e1008106. doi: 10.1371/journal.pcbi.1008106 (PMC7449454; doi:10.1371/journal.pcbi.1008106)
Supplement: S1 Table — To our knowledge, the association rate for ciprofloxacin has not been determined directly. Because the values of the ratio of dissociation rate kr and association rate kf, KD, diverge by more than an order of magnitude in the literature, we chose to fit the association rate kf as a free parameter in our model while constraining KD to remain within the published range (the resulting value of kf is given together with other fitted parameters in S2 Table). (DOCX) [file pcbi.1008106.s015.docx]

| **Antibiotic** | **Target** | **Parameter** | **Value** | **References** |
| --- | --- | --- | --- | --- |
| Ciprofloxacin | Gyrase | Copy #/cell | Gyrase: 50-100 functional tetramers | (1, 2) |
|  |  | Unbinding rate *k_r_* | 3⋅10^-4^ sec^-1^ | (3) |
|  |  | *K_D_* (*k_r_/ k_f_)* | 10^-5.6^  10^-5.7^  10^-5.9^  10^-7.5^ *(lower boundary according to authors)* | (4)  (5)  (6)  (3) |
| Ampicillin | Penicillin binding proteins | Copy #/cell | ~ 2500±120 | (7, 8) |
|  |  | Binding rate *k_f_* | 130 | (9, 10) |
|  |  | Deacetylation rate *k_a_* | 10^-4^ | (10) |

1. Maier T*, et al.* (2011) Quantification of mRNA and protein and integration with protein turnover in a bacterium. *Mol Syst Biol* 7:511.

2. Malmstrom J*, et al.* (2009) Proteome-wide cellular protein concentrations of the human pathogen Leptospira interrogans. *Nature* 460(7256):762-765.

3. Kampranis SC & Maxwell A (1998) The DNA gyrase-quinolone complex. ATP hydrolysis and the mechanism of DNA cleavage. *J Biol Chem* 273(35):22615-22626.

4. Siporin C, Heifetz CL, & Domagala JM (1990) *The New generation of quinolones* (M. Dekker, New York) pp xii, 347 p., 342 p. of plates.

5. Shen LL*, et al.* (1989) Mechanism of inhibition of DNA gyrase by quinolone antibacterials: a cooperative drug--DNA binding model. *Biochemistry* 28(9):3886-3894.

6. Jungkind DL & American Society for Microbiology Eastern Pennsylvania Branch (1995) *Antimicrobial resistance a crisis in health care* (Plenum Press, New York etc.) pp X, 248 S.

7. Dougherty TJ, Kennedy K, Kessler RE, & Pucci MJ (1996) Direct quantitation of the number of individual penicillin-binding proteins per cell in Escherichia coli. *J Bacteriol* 178(21):6110-6115.

8. Spratt BG (1975) Distinct penicillin binding proteins involved in the division, elongation, and shape of Escherichia coli K12. *Proc Natl Acad Sci U S A* 72(8):2999-3003.

9. Abel Zur Wiesch P, Clarelli F, & Cohen T (2017) Using Chemical Reaction Kinetics to Predict Optimal Antibiotic Treatment Strategies. *PLoS Comput Biol* 13(1):e1005321.

10. Terrak M*, et al.* (1999) The catalytic, glycosyl transferase and acyl transferase modules of the cell wall peptidoglycan-polymerizing penicillin-binding protein 1b of Escherichia coli. *Mol Microbiol* 34(2):350-364.
