# Supplementary material for: Drug-target binding quantitatively predicts optimal antibiotic dose levels in quinolones
Source: PLoS Comput Biol. 2020 Aug 14;16(8):e1008106. doi: 10.1371/journal.pcbi.1008106 (PMC7449454; doi:10.1371/journal.pcbi.1008106)
Supplement: S2 Table — Fig 3A shows the model fit and Fig 3B shows the resulting functions for the replication rate r(x) and the death rate δ(x) as a function of the number of bound targets. (DOCX) [file pcbi.1008106.s016.docx]

| **Parameter** | **Value** | **Unit of measure** | **Explanation** |
| --- | --- | --- | --- |
| *k_f_* | 3.210⋅10^3^ | M^-1^sec^-1^ | Binding rate |
| *r_0_* | 2.416·10^-4^ | sec^-1^ | Maximum replication WT |
| *a_1_* | 2.441·10^-4^ | sec^-1^ | Coefficient of r(x) |
| *b_1_* | 0.0459 | - | Coefficient of r(x) |
| *c_1_* | -2.478·10^-6^ | sec^-1^ | Coefficient of r(x) |
| *δ_m_* | 0.0058 | sec^-1^ | Maximum death rate |
| *a_2_* | 2.46·10^-5^ | sec^-1^ | Coefficient of δ(x) |
| *b_2_* | 0.0547 | - | Coefficient of δ(x) |
| *c_2_* | -2.46·10^-5^ | sec^-1^ | Coefficient of δ(x) |
