# Supplementary material for: Drug-target binding quantitatively predicts optimal antibiotic dose levels in quinolones
Source: PLoS Comput Biol. 2020 Aug 14;16(8):e1008106. doi: 10.1371/journal.pcbi.1008106 (PMC7449454; doi:10.1371/journal.pcbi.1008106)
Supplement: S3 Table — (m) indicates mild overexpression, (s) indicates strong overexpression. Columns headed GyrA and GyrB show the experimentally determined overexpression as fold expression compared to the wild type. The columns headed GyrA2B2 show the estimated tetramer levels resulting from each measurement. For the GyrA2B2 tetramer estimation, we sampled 104 sets association and dissociation rates from a uniform distribution within their reported limits (Latin hypercube approach). We report the standard deviation for each estimate. We give summary estimates in the last row of the table. (DOCX) [file pcbi.1008106.s017.docx]

| **GyrA (m)** | **GyrB (m)** | **GyrA_2_B_2_ (m)** | **GyrA (s)** | **GyrB (s)** | **GyrA_2_B_2_ (s)** |
| --- | --- | --- | --- | --- | --- |
| 1.67 | 1.85 | 1.7136 ± 0.0004 | 2.43 | 3.46 | 2.5017 ± 0.001 |
| 1.09 | 1.20 | 1.1121 ± 0.002 | 1.44 | 1.68 | 1.4803 ± 0.0004 |
| 1.11 | 1.21 | 1.1673 ± 0.0002 | 1.56 | 1.94 | 1.6024 ± 0.0005 |
| 2.04 | 2.99 | 2.1004 ± 0.0009 | 3.36 | 6.65 | 3.4706 ± 0.0016 |
| 1.16 | 1.26 | 1.1802 ± 0.0002 | 1.77 | 2.29 | 1.8229 ± 0.0007 |
| 1.25 | 1.51 | 1.2797 ± 0.0004 | 1.98 | 3.04 | 2.0401 ± 0.0008 |
| Mean + IC 95% | | 1.43 (1.119 – 1.81) | Mean + IC 95% | | 2.15 (1.73 – 2.87) |
