# Supplementary material for: Drug-target binding quantitatively predicts optimal antibiotic dose levels in quinolones
Source: PLoS Comput Biol. 2020 Aug 14;16(8):e1008106. doi: 10.1371/journal.pcbi.1008106 (PMC7449454; doi:10.1371/journal.pcbi.1008106)
Supplement: S4 Table — Fig 5B shows the resulting death rate δ(x) as an exponential function of the number of bound targets δ(x) = a3 eb3x + c3. (DOCX) [file pcbi.1008106.s018.docx]

| **Parameter** | **Value** | **Unit of measure** | **Explanation** |
| --- | --- | --- | --- |
| *r_0_* | 4.664·10^-4^ | sec^-1^ | Maximum replication WT |
| *a_3_* | 1.075·10^-5^ | sec^-1^ | Coefficient of δ(x) |
| *b_3_* | 0.023 | - | Coefficient of δ(x) |
| *c_3_* | -1.075·10^-6^ | sec^-1^ | Coefficient of δ(x) |
| *δ_m_* | 0.0034 | sec^-1^ | Maximum death rate |
