# Supplementary material for: Drug-target binding quantitatively predicts optimal antibiotic dose levels in quinolones
Source: PLoS Comput Biol. 2020 Aug 14;16(8):e1008106. doi: 10.1371/journal.pcbi.1008106 (PMC7449454; doi:10.1371/journal.pcbi.1008106)
Supplement: S5 Table — The parameters were obtained from [60]. (DOCX) [file pcbi.1008106.s019.docx]

| **Parameter** | **Value** | **Description** |
| --- | --- | --- |
| *V*_1_ | 12.5 L | Apparent volume – compartment 1 “plasma” |
| *V*_2_ | 6.15 L | Apparent volume – compartment 2 “tissue” |
| *k*_10_ | 1.16 h^-1^ | Elimination rate |
| *k*_12_ | 0.66 h^-1^ | Intercompartment transfer rate (1 to 2) |
| *k*_21_ | 1.33 h^-1^ | Intercompartment transfer rate (2 to 1) |
| *τ* | 0-24 h | Daily duration of infusion |
