# Supplementary material for: Drug-target binding quantitatively predicts optimal antibiotic dose levels in quinolones
Source: PLoS Comput Biol. 2020 Aug 14;16(8):e1008106. doi: 10.1371/journal.pcbi.1008106 (PMC7449454; doi:10.1371/journal.pcbi.1008106)
Supplement: S1 Text — (DOCX) [file pcbi.1008106.s020.docx]

Drug-target binding models such as COMBAT are better suited than traditional pharmacodynamic models to accurately reflect drug concentrations that fluctuate over time [1, 2]. COMBAT can be coupled with any time-concentration profile for example concentrations in patients predicted by pharmacokinetic models. Here, we use a two compartment pharmacokinetic model to predict the antibiotic concentration in the infected target tissue (Fig. S13). We model two different modes of administration of the same daily drug dose: single short (5 min; mimicking bolus injection) and continuous infusions. As an example, we choose ampicillin (2 g per day) and obtained realistic parameters from patient data [3] to inform the pharmacokinetic model (Tab. S5).

The pharmacokinetic model is described by

$\frac{dC_{p}\left( t \right)}{dt}=q_{0}\cdot\theta\left( \tau-t \right)-k_{10}C_{p}\left( t \right)-k_{12}C_{p}\left( t \right)+k_{21}C_{t}\left( t \right)$ (S1)

$\frac{dC_{t}\left( t \right)}{dt}=+k_{12}C_{p}\left( t \right)-k_{21}C_{t}\left( t \right)$ (S2)

Where *C*_p_ is the drug concentration in plasma and *C*_t_ the drug concentration in the tissue compartment. $q_{0}=\frac{D_{0}}{V_{1}\tau} \left( \frac{mg}{L\cdot sec} \right)$ describes the drug administration, $k_{10}$ is the elimination rate, and $\tau$ is the infusion time. The changing drug concentration over time is shown in Fig. S14a.

Since the total volume of infected tissue is unknown, our model of the infection process is normalized to V_inf_ = 1 mL, where the drug concentration is given by *C*_t_(t). Here, the amount of drug molecules is $A_{inf}\left( t \right)={\frac{C_{t}\left( t \right)}{\mu_{a}}N}_{A}V_{inf}$ (where $\mu_{a}$ is the molar mass of ampicillin and N_A_ the Avogadro number). We therefore can neglect the effect of the association and dissociation rate on *C*_t_(t), i.e. equation S1 and equation S2 are independent on the solution of equation S3.

The effect of the determined fluctuating drug concentration on pathogen growth is then determined by

$\frac{\partial B\left( x,t \right)}{\partial t}=\overset{Association term}{\overbrace{\frac{\partial}{\partial x}\left( \hat{k}_{f}A_{inf}\left( t \right)\left( \theta-x \right)B\left( x,t \right) \right)}-}\overset{Dissociation term}{\overbrace{\frac{\partial}{\partial x}\left( k_{r}xB\left( x,t \right) \right)}}$ (S3)

$-\overset{Replication and its effects on binding}{\overbrace{r(x)B(x,t)F_{lim}(t)+S_{B}(x,t)F_{lim}(t)}}\overset{Death}{-\overbrace{\delta(x)B(x,t)}}$

(see equation 2). While the high drug concentrations of the single daily dosing regimen have a strong, short effect on bacterial numbers in the target tissue, the average bacterial load increases (Fig. S14b). In contrast, after a short initial period of growth, the continuous infusion of ampicillin leads to a steady decrease of bacterial numbers over time (Fig. S14b).

**References**

1. Clarelli F, Liang J, Martinecz A, Heiland I, & Abel Zur Wiesch P (2020) Multi-scale modeling of drug binding kinetics to predict drug efficacy. Cell Mol Life Sci 77(3):381-394.

2. Tonge PJ (2018) Drug-Target Kinetics in Drug Discovery. ACS Chem Neurosci 9(1):29-39.

3. Ripa S, Ferrante L, & Prenna M (1990) Pharmacokinetics of sulbactam/ampicillin in humans after intravenous and intramuscular injection. Chemotherapy 36(3):185-192.
